# Further correction: Expanding medicinal chemistry into 3D space: metallofragments as 3D scaffolds for fragment-based drug discovery

**DOI:** 10.1039/d3sc90164e

**Published:** 2023-09-01

**Authors:** Christine N. Morrison, Kathleen E. Prosser, Ryjul W. Stokes, Anna Cordes, Nils Metzler-Nolte, Seth M. Cohen

**Affiliations:** a Department of Chemistry and Biochemistry, University of California San Diego La Jolla CA 92093 USA scohen@ucsd.edu; b Lehrstuhl für Anorganische Chemie 1, Bioanorganische Chemie, Ruhr-Universität Bochum, Universitätsstraße 150 44801 Bochum Germany

## Abstract

Further correction for ‘Expanding medicinal chemistry into 3D space: metallofragments as 3D scaffolds for fragment-based drug discovery’ by Christine N. Morrison *et al.*, *Chem. Sci.*, 2020, **11**, 1216–1225, https://doi.org/10.1039/C9SC05586J.

The authors regret that in the original article, inhibitory values reported for some metallofragments were not reproducible. Unfortunately, stock solutions of reportedly active Ru-arene-based metallofragments (‘class J’) were found to decompose when stored for extended periods of time in DMSO, which resulted in inconsistent inhibition values. The authors maintain that the central conclusions of the paper are accurate and the utility of three-dimensional metal complexes for fragment-based drug discovery has merit.

In the original article, ‘class J’ metallofragments comprise a set of Ru-arene derivatives (Fig. 1). Some of these metallofragments are reported as inhibiting the polymerase acidic N-terminal (PAN) endonuclease domain from the H1N1 influenza A virus and New Delhi metallo-β-lactamase-1 (NDM-1). However, re-evaluation of these compounds against PAN and NDM-1 failed to reproduce our original inhibition results when fresh *versus* ‘aged’ stock solutions were used, indicating a problem with the data concerning these compounds in our original study.

**Fig. 1:**
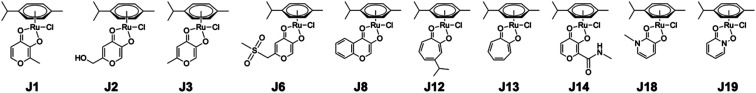
Chemical structures of Ru-arene compounds that comprise the original class J metallofragments. Compounds J1, J13, and J19 were re-examined for their stability and inhibitory activity in this correction.

Once we became aware of the potential issue, we performed additional experiments to determine the stability of class J compounds. ^1^H NMR analysis was performed on representative compounds stored in deuterated DMSO-d_6_. Compounds J1, J13, and J19 were prepared as 50 mM DMSO-d_6_ solutions and their ^1^H NMR spectra were collected at 30 min (shortly after preparation), 24 h, and 48 h (Fig. 2). The spectra showed that the compounds decompose (or undergo ligand exchange) over time in DMSO-d_6_, primarily *via* ligand exchange with DMSO. The observed ligand exchange is consistent with a previous study of these compounds (*Chem.–Eur. J.* 2013, **19**, 14768–14772), which was also cited in our original publication (as ref. 46). The three compounds show greatly varying degrees of decomposition, with J1 showing the greatest spectral change, followed by J19, and J13, the latter of which shows quite minimal change over the 48 h time period. This suggest that the stability of these metallofragments (and potential utility as 3-dimensional scaffolds) may vary depending on the nature of the bidentate ligand. By contrast, storage of compounds J1, J13, and J19 at 50 mM in acetone-d_6_ showed no change over the same 30 min, 24 h, and 48 h time periods (Fig. 2).

**Fig. 2:**
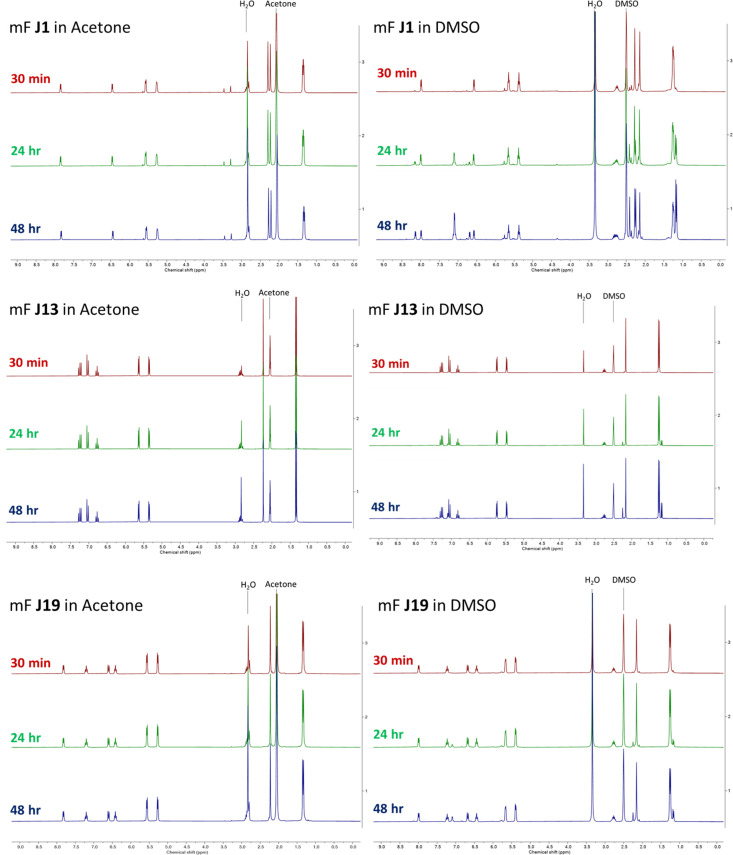
^1^H NMR analysis of representative class J metallofragments stored as 50 mM solutions in acetone-d_6_ (left) and DMSO-d_6_ (right). All three compounds are stable in acetone-d_6_, but only compound J13 shows long term stability in DMSO-d_6_.

Based on the stability findings, it was unsurprising to find that some of the inhibition data associated with the class J metallofragments were not reproducible. Re-evaluation of J1, J13, and J19 against PAN endonuclease and NDM-1 using freshly prepared DMSO stock solutions was performed in triplicate in three independent experiments. Appropriate positive (fully-active enzyme) and negative (absence of enzyme) controls were performed with each assay. Inhibitory activity was measured at a metallofragment concentration of 200 μM. Compounds J1, J13, and J19 were tested in the enzymatic assays as fresh DMSO stocks, as well as stock solutions that had been stored at room temperature for 24 and 48 h.

Unfortunately, the repeated assay experiments revealed discrepancies in the originally reported inhibition values for J1, J13, and J19 against PAN endonuclease assay. Freshly prepared samples of J1, J13, and J19 all showed substantial differences from their originally reported percent inhibition values (Table 1). In addition, compounds J1 and J19 showed greater activity as the stock solutions aged, nominally consistent with the NMR stability studies (Fig. 2). Consistent with its greater stability in DMSO, compound J13 produced generally consistent inhibition values for fresh and aged stock solutions.

**Table 1:** Reported (original) and corrected (this correction) percent inhibition values of class J metallofragments against PAN endonuclease at an inhibitor concentration of 200 μM in DMSO. Freshly prepared DMSO stock solutions were evaluated, along with stocks that had been aged 24 or 48 h at room temperature

Compound	J1	J13	J19
Reported	25 ± 6	44 ± 5	65 ± 5
Corrected (fresh)	8 ± 14	65 ± 7	30 ± 12
Corrected (24 h)	16 ± 12	68 ± 7	37 ± 11
Corrected (48 h)	19 ± 9	67 ± 8	48 ± 6

For NDM-1, freshly prepared samples of J1, J13, and J19 all showed even greater discrepancies (when compared to PAN) from their originally reported percent inhibition values (Table 2). The originally reported data indicate that all three compounds show substantial inhibition activity (>70%, Table 2); however, the re-evaluated, inhibition data show that the compounds are much less active than initially determined. Compounds J1 and J19 showed a notable decrease in activity upon aging in DMSO. Only compound J13 exhibited significant inhibition of NDM-1 (>50%) over all three time points, again consistent with its greater stability in DMSO.

**Table 2:** Reported (original) and corrected (this correction) percent inhibition values of class J metallofragments against NDM-1 at an inhibitor concentration of 200 μM in DMSO. Freshly prepared DMSO stock solutions were evaluated, along with stocks that had been aged 24 or 48 h at room temperature

Compound	J1	J13	J19
Reported	81 ± 1	77 ± 2	98 ± 1
Corrected (fresh)	27 ± 5	64 ± 6	42 ± 3
Corrected (24 h)	5 ± 9	51 ± 2	19 ± 3
Corrected (48 h)	6 ± 12	57 ± 15	27 ± 14

Taken together, the authors regret that the inhibitory data associated with class J metallofragments are not readily reproducible, likely because of decomposition of these compounds upon extended storage in DMSO. The authors maintain that 3-dimensional metallofragments represent a useful new line of inquiry for fragment-based drug discovery (FBDD) and our ongoing studies seek to further test this hypothesis. However, the authors acknowledge that metallofragments may pose unique challenges, specifically with respect to stability, that must be carefully considered and controlled for when using them in FBDD campaigns.

The authors would like to take this opportunity to thank the readers who alerted them to the concerns regarding the inhibitory activities and allowed them to reinvestigate. Both the authors and the Royal Society of Chemistry appreciate their support. The authors also thank Hyeonglim Seo (U.C. San Diego), as well as Dann D. Rivera, Thomas Smisek, and Walter Fast (U.T. Austin) for their efforts in acquiring the additional data reported here and their assistance with preparing this correction.

The Royal Society of Chemistry apologises for these errors and any consequent inconvenience to authors and readers.

## Supplementary Material

